# Repeated post-exercise administration with a mixture of leucine and glucose alters the plasma amino acid profile in Standardbred trotters

**DOI:** 10.1186/1751-0147-54-7

**Published:** 2012-02-01

**Authors:** Katarina EA Nostell, Birgitta Essén-Gustavsson, Johan T Bröjer

**Affiliations:** 1 Department of Clinical Sciences, Swedish University of Agricultural Sciences, Box 7054, S-750 07 Uppsala, Sweden

**Keywords:** horse, exercise, amino acid, leucine, glucose, insulin

## Abstract

**Background:**

The branched chain amino acid leucine is a potent stimulator of insulin secretion. Used in combination with glucose it can increase the insulin response and the post exercise re-synthesis of glycogen in man. Decreased plasma amino acid concentrations have been reported after intravenous or per oral administration of leucine in man as well as after a single per oral dose in horses. In man, a negative correlation between the insulin response and the concentrations of isoleucine, valine and methionine have been shown but results from horses are lacking. This study aims to determine the effect of repeated per oral administration with a mixture of glucose and leucine on the free amino acid profile and the insulin response in horses after glycogen-depleting exercise.

**Methods:**

In a crossover design, after a glycogen depleting exercise, twelve Standardbred trotters received either repeated oral boluses of glucose, 1 g/kg body weight (BW) at 0, 2 and 4 h with addition of leucine 0.1 g/kg BW at 0 and 4 h (GLU+LEU), or repeated boluses of water at 0, 2 and 4 h (CON). Blood samples for analysis of glucose, insulin and amino acid concentrations were collected prior to exercise and over a 6 h post-exercise period. A mixed model approach was used for the statistical analyses.

**Results:**

Plasma leucine, isoleucine, valine, tyrosine and phenylalanine concentrations increased after exercise. Post-exercise serum glucose and plasma insulin response were significantly higher in the GLU+LEU treatment compared to the CON treatment. Plasma leucine concentrations increased after supplementation. During the post-exercise period isoleucine, valine and methionine concentrations decreased in both treatments but were significantly lower in the GLU+LEU treatment. There was no correlation between the insulin response and the response in plasma leucine, isoleucine, valine and methionine.

**Conclusions:**

Repeated post-exercise administration with a mixture of leucine and glucose caused a marked insulin response and altered the plasma amino acid profile in horses in a similar manner as described in man. However, the decreases seen in plasma amino acids in horses seem to be related more to an effect of leucine and not to the insulin response as seen in man.

## Introduction

Leucine is one of three branched chain amino acids (BCAA) and a potent stimulator of insulin secretion, which is mediated by oxidative decarboxylation and allosteric activation of glutamate dehydrogenase [1-3]. The metabolically linked secondary signals that lead to insulin release have not yet been established. The combination of carbohydrates and protein or amino acids has been shown to potentiate the insulin response and increase the post exercise re-synthesis of muscle glycogen in man [4,5]. In horses, per oral administration of leucine and glucose post exercise has been shown to induce an insulin response equal to the one seen after administration of intravenous glucose [6,7]. Contrary to man, the post-exercise re-synthesis of muscle glycogen is not increased in horses [7].

There are indications that supplementation with leucine not only affects glucose metabolism but also protein metabolism. In humans, intravenous administration of leucine [8-10] or per oral administration of BCAA [11] caused decreased concentrations of the other branched chain amino acids (isoleucine and valine) and the aromatic amino acids (phenylalanine, tyrosine and tryptophan) as well as some of the other plasma amino acids. Decreased plasma amino acid concentrations have also been reported after per oral supplementation with leucine as well as BCAA before and after exercise [12,13]. A recently published study in horses showed similar effects on the BCAA profile after a single gastric gavage of leucine in the early recovery period after glycogen depleting treadmill exercise [6].

Endogenous and exogenous insulin is known to be effective in lowering plasma amino acid concentrations in humans [14,15]. This could be related to the fact that insulin has been shown to stimulate the transportation of amino acids into cells as well as reducing the release of free amino acids in plasma [14,16]. It is therefore possible that the decrease seen in amino acid concentration, at least to some extent, is related to the increased insulinaemic response. A negative correlation between the insulin response and the plasma amino acid concentration has also been shown in man, where the decrease was most pronounced for isoleucine, valine and methionine [17]. If a similar correlation exists in horses has not been shown.

The aim of the present study was to investigate the effect of repeated per oral administration of a mixture of glucose and leucine on the free amino acid profile in horses after glycogen-depleting exercise. Another aim was to study if amino acid concentrations showed any relationship to the insulin response as reported in humans.

## Materials and methods

Enrolment in this study was done in parallel with a study with the purpose to study the insulin response and glycogen resynthesis after post-exercise supplementation with leucine and glucose [7]. For more detailed information regarding horses, diet and exercise, see [7]. The study (C338/8) was sanctioned by the Ethical Committee for Animal Experiments, Uppsala, Sweden.

### Horses and diet

Twelve race conditioned Standardbred trotters (7 geldings and 5 mares; body weight 406 - 536 kg; age 4 - 9 years) accustomed to perform intermittent exercise on a slope were included in this study. The horses were feed a standardised diet consisting of 6.5 - 8.0 kg haylage (10.3% CP and 9 MJ metabolisable energy per kg on a MD basis) and 3.3-4.8 kg of a commercial pelleted concentrate (11.9% CP and 11 MJ metabolisable energy per kg on a DM basis, Krafft, Lantmännen Krafft AB, Falkenberg, Sweden) and had free access to water and a mineral block. A diet acclimation period of three weeks was used before the start of the experiment.

### Exercise test

The horses performed a glycogen depleting field exercise test described by Bröjer *et al. *[18]. In brief, the test included a 4000 m warm up at a slow trot, followed by 7 repeated 500 m up-hill intervals at a speed of 9 m/s and a down hill walk between each interval. After finishing the last interval the horses trotted slowly back to the stable. The exercise test was performed in pairs where the horses were matched according to their age and training status. The horses were allowed free access to water but feed was withheld 9 h prior to the exercise test.

### Experimental protocol

The study was performed as a random crossover design with two different oral treatments, glucose and leucine (GLU+LEU) or control (CON), administered during the early post-exercise period following glycogen-depleting exercise. Fifteen minutes after completion of exercise (time 0), the horses received either the GLU+LEU or the CON treatment by gastric gavage. The treatments were randomly allocated in each pair of horses. The GLU+LEU treatment consisted of 3 boluses (glucose 1 g/kg body weight (BW) as a 10% solution) at 0, 2 and 4 h during the post-exercise period. In addition, 0.1 g/kg BW of leucine was added to the boluses of glucose at 0 and 4 h. The CON treatment consisted of an equivalent volume of water that was administered at 0, 2 and 4 h during the post-exercise period. Feed and water was withheld until the end of the sampling period. Each treatment was separated by a 6 wk washout period.

### Heart frequency and blood collection

Heart frequency was registered continuously before, during and immediately after completion of the exercise test using a pulse meter (Polar Electron OY, Kempele, Finland).

Intravenous catheters (Intranule, Vygon, 14 gauge, 10.5 cm, Ecouen, France) were introduced under local anaesthesia (Lidocain, 2%, AstraZenicaAB, Sodertälje, Sweden) into one of the jugular veins. Blood samples were collected before and immediately after exercise, at time 0 (immediately after first oral administration), every 15 min during the first hour, and every 30 min during the following 5 h. Samples for analysis of plasma amino acids and insulin were collected in heparinised evacuated tubes and in tubes without additive for analysis of serum glucose (Vacutainer, BD, Belliver Industrial Estate, Plymouth, UK). All samples were centrifuged for 15 min (2700 *g*), plasma and serum subsequently harvested and stored for 3 d at -20°C and then transferred to a -80°C until analysis.

### Sample analysis

Serum glucose concentrations were measured using an automated analyser (Architect ci8200, Abbott Scandinavia AB Diagnostics, Solna, Sweden). Plasma insulin concentrations were measured in duplicate with a commercial equine-optimized ELISA (Insulin Equine ELISA, Mercodia AB, Uppsala, Sweden) validated for use in horses [19]. The results from the assay are reported in ng/l. The intra assay coefficient of variation (CV) for the insulin assay varied between 1.9 and 4.8% for the individual runs and the inter assay CV was 8.0%.

Plasma free amino acids were measured after precipitation of proteins with a 1:10 dilution with 5% trichloroacetic acid followed by centrifugation at 2700 *g*. Supernatants were collected and stored at -80°C until the assays were performed. The content of amino acids (μmol/l) was measured with reversed-phase HPLC using a 5 μm 150 × 3.9 mm C18 column (Resolve™ C18 90 Å) according to [20]. Prior to injection, precolumn derivatisation of samples was performed with 0.04 M *o*-phthalaldehyde.

### Statistical methods

A Student's paired t-test was used to evaluate the change in plasma amino acids before and after exercise (*P *≤ 0.05). A mixed model approach was used for the statistical analyses. Data were analysed using the mixed procedure in the SAS (2008) system (SAS Institute Inc., Cary, NC, USA). Since time points were not equidistant, a special power covariance structure was used to model the within-horse covariance's over time. The model included treatment, time, and the interaction between these. Specific questions on comparisons between time points were answered by post-hoc tests adjusted for multiplicity using Tukey's method. Statistical significance was set at *P *≤ 0.05. Values are presented as mean ± SEM. Data on the combined effects of work and treatment with four data points per horse in a change-over design, were analysed as a mixed linear model with treatment, work and the interaction between these as fixed factors and using horse as a random factor. The area under the curve (AUC) during the 360 min post-exercise period for glucose, insulin, leucine, isoleucine, valine and methionine was calculated with a computer software program (SigmaPlot software version 11, SPSS Science, USA) using the trapezoidal approximation. For amino acids that significantly changed after supplementation with GLU+LEU, correlations between the insulin response (AUC) and amino acid response (AUC) over 360 min were calculated, using the Pearson's correlation test.

## Results

One horse became lame during the exercise test and was excluded leaving 11 horses in the study. The overall mean heart rate at the end of the intervals for the two experimental periods was 210 ± 2 and 211 ± 1 beats/min respectively.

### Serum glucose and plasma insulin response

**Figure 1:**
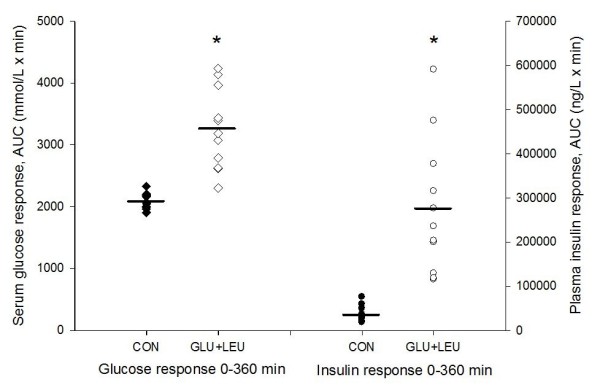
**Serum glucose and plasma insulin response over the 360 min post-exercise period**. Scatterplot showing results of individual serum glucose and plasma insulin response expressed as area under the curve (AUC) during the 360 min post-exercise period in the two different treatments (GLU+LEU = oral glucose and leucine; CON = control) in 11 horses. Serum glucose response CON treatment, filled diamond; GLU+LEU treatment, unfilled diamond; plasma insulin response CON treatment, filled circle; GLU+LEU treatment, unfilled circle. The horizontal line represents the mean response. *Value in the GLU+LEU treatment differs from the control treatment (*P *< 0.05).

The concentrations of serum glucose and plasma insulin immediately after exercise and during the post-exercise period have been reported elsewhere [7]. Serum glucose and plasma insulin concentrations increased immediately after exercise in both treatment groups with no significant differences between the groups [7]. The individual serum glucose response (AUC) is shown in Figure 1. The mean glucose response (AUC) was significantly higher in the GLU+LEU treatment compared to the CON treatment. The mean insulin response (AUC) for the entire post exercise period was markedly increased in the GLU+LEU treatment compared to the CON treatment, but with large individual variations in insulin response (Figure 1).

### Plasma amino acid concentration

**Figure 2:**
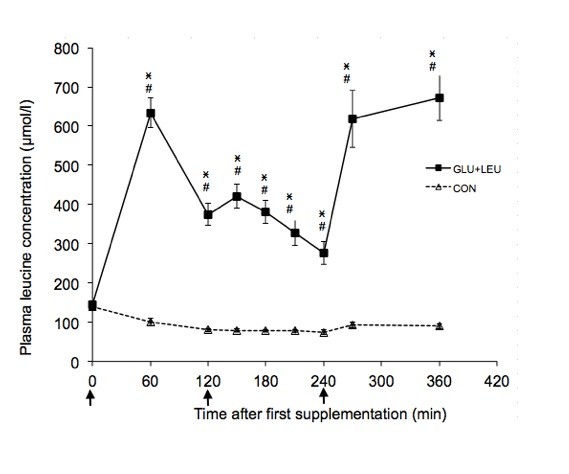
**Plasma concentrations of leucine, isoleucine, valine and methionine during the 360 min post-exercise period**. Mean concentrations ± SE for plasma leucine (figure 2, n = 11), isoleucine (figure 3, n = 11), valine (figure 4, n = 11) and methionine (figure 5, n = 6) during the 360 min post-exercise period for different treatments (GLU+LEU = oral glucose and leucine; CON = control). Arrows indicate oral administration of glucose and leucine (time points 0 and 240 min) or only glucose (time point 120 min). ^# ^Within a treatment, value differs significantly (*P *< 0.05) differs from resting values. *Within a time point, value for GLU+LEU treatment differs significantly (*P *< 0.05) from the value for the CON treatment.

**Figure 3:**
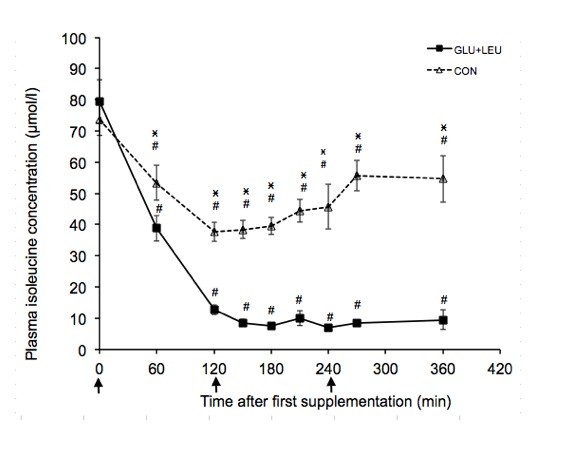
**Plasma concentrations of leucine, isoleucine, valine and methionine during the 360 min post-exercise period**. Mean concentrations ± SE for plasma leucine (figure 2, n = 11), isoleucine (figure 3, n = 11), valine (figure 4, n = 11) and methionine (figure 5, n = 6) during the 360 min post-exercise period for different treatments (GLU+LEU = oral glucose and leucine; CON = control). Arrows indicate oral administration of glucose and leucine (time points 0 and 240 min) or only glucose (time point 120 min). ^# ^Within a treatment, value differs significantly (*P *< 0.05) differs from resting values. *Within a time point, value for GLU+LEU treatment differs significantly (*P *< 0.05) from the value for the CON treatment.

**Figure 4:**
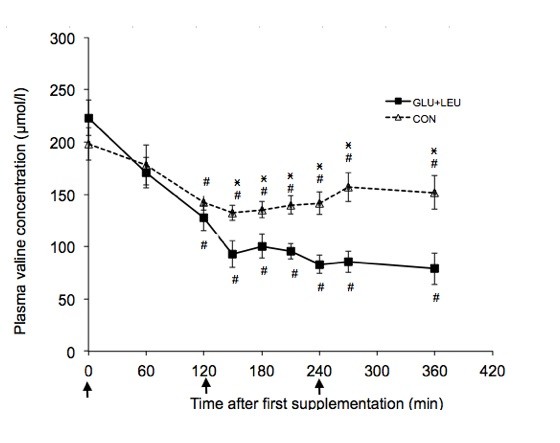
**Plasma concentrations of leucine, isoleucine, valine and methionine during the 360 min post-exercise period**. Mean concentrations ± SE for plasma leucine (figure 2, n = 11), isoleucine (figure 3, n = 11), valine (figure 4, n = 11) and methionine (figure 5, n = 6) during the 360 min post-exercise period for different treatments (GLU+LEU = oral glucose and leucine; CON = control). Arrows indicate oral administration of glucose and leucine (time points 0 and 240 min) or only glucose (time point 120 min). ^# ^Within a treatment, value differs significantly (*P *< 0.05) differs from resting values. *Within a time point, value for GLU+LEU treatment differs significantly (*P *< 0.05) from the value for the CON treatment.

**Figure 5:**
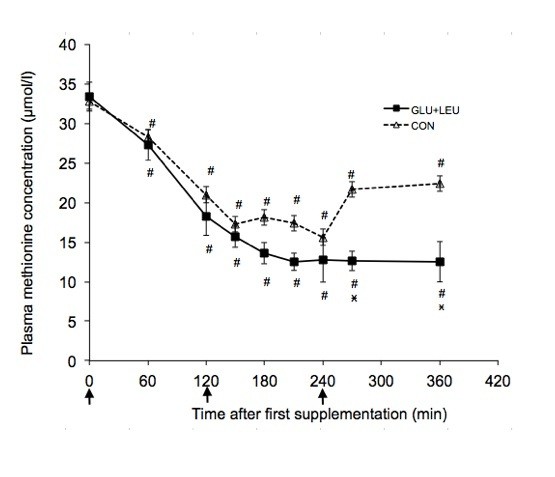
**Plasma concentrations of leucine, isoleucine, valine and methionine during the 360 min post-exercise period**. Mean concentrations ± SE for plasma leucine (figure 2, n = 11), isoleucine (figure 3, n = 11), valine (figure 4, n = 11) and methionine (figure 5, n = 6) during the 360 min post-exercise period for different treatments (GLU+LEU = oral glucose and leucine; CON = control). Arrows indicate oral administration of glucose and leucine (time points 0 and 240 min) or only glucose (time point 120 min). ^# ^Within a treatment, value differs significantly (*P *< 0.05) differs from resting values. *Within a time point, value for GLU+LEU treatment differs significantly (*P *< 0.05) from the value for the CON treatment.

Exercise increased the plasma concentrations of BCAA, aromatic amino acids and glutamate and decreased the concentration of glutamine (Table 1). There was no significant effect of exercise on the other plasma amino acids concentrations. In the post-exercise period there was a significant effect of time (*P *< 0.05) on the plasma concentrations of leucine, isoleucine, valine, phenylalanine, tryptophan, tyrosine, threonine, methionine, glutamine, glutamate, histidine, and serine. There was a significant effect of supplement on the plasma concentrations of leucine (Figure 2), isoleucine (Figure 3), valine (Figure 4), and methionine (Figure 5) and a significant interaction of time and supplement on the plasma concentrations of leucine, isoleucine, valine, and methionine (*P *≤ 0.05). Plasma concentrations of leucine rose markedly after supplementation (Figure 2). Plasma concentrations of isoleucine, valine and methionine were significantly decreased in the GLU+LEU treatment compared to the CON treatment (Figure 3, 4, 5). Plasma concentrations for the other amino acids were not significantly different between treatments (Table 2). There was no correlation between the insulin response (AUC 360 min) and the response (AUC 360 min) of leucine (r = 0.19), isoleucine (r = 0.53), valine (r = 0.43) and methionine (r = 0.10) during the post-exercise period.

**Table 1:** Mean plasma amino acid concentrations (μmol/l) before and after exercise

	GLU+ LEU		CON	
	Before	After exercise	Before	After exercise
Leucine	84 ± 7	128 ± 13*	87 ± 7	136 ± 7*
Isoleucine	58 ± 5	77 ± 8*	57 ± 5	71 ± 5*
Valine	168 ± 20	203 ± 23*	171 ± 20	191 ± 14*
Tyrosine	61 ± 5	73 ± 7*	58 ± 4	73 ± 4*
Phenylalanine	60 ± 5	84 ± 6*	63 ± 4	81 ± 7*
Glutamate	24 ± 2	45 ± 9*	26 ± 2	36 ± 3*
Glutamine	255 ± 30	204 ± 24*	248 ± 18	211 ± 21*
Glycine	454 ± 54	434 ± 59	419 ± 30	433 ± 33
Methionine	22 ± 2	28 ± 2	25 ± 2	34 ± 3
Serine	34 ± 3	32 ± 3	34 ± 3	34 ± 4
Threonine	137 ± 16	133 ± 14	126 ± 13	134 ± 14
Histidine	69 ± 6	58 ± 4	70 ± 6	64 ± 5
Metyl-histidine	33 ± 4	28 ± 3	36 ± 6	31 ± 5
Tryptophan	89 ± 5	92 ± 7	88 ± 7	99 ± 10

Mean values ± SEM are shown for plasma amino acid (μmol/l) concentrations before and after two glycogen depleting field exercise tests. The exercise tests were performed by 11 horses before they were given two different oral treatments during the early post-exercise period, either glucose and leucine (GLU+LEU) or water (CON). *Significantly different from rest.

**Table 2:** Mean plasma amino acid concentrations over the 360 min recovery period

Plasma amino acid concentration		Time after first supplementation (min)								
(μmol/l)	Treatment	0	60	120	150	180	210	240	270	360
**Glutamate**	GLU+LEU	35 ± 2	28 ± 4	22 ± 2	25 ± 5	20 ± 2	22 ± 1	23 ± 3	21 ± 2	21 ± 2
	CON	32 ± 2	27 ± 1	22 ± 2	22 ± 2	20 ± 2	20 ± 2	19 ± 1	20 ± 3	21 ± 2
**Serin**	GLU+LEU	36 ± 2	26 ± 2	23 ± 2	26 ± 3	23 ± 2	23 ± 1	21 ± 2	24 ± 2	25 ± 3
	CON	37 ± 3	29 ± 2	25 ± 2	23 ± 2	23 ± 2	22 ± 2	24 ± 3	27 ± 4	27 ± 2
**Glutamine**	GLU+LEU	230 ± 20	180 ± 10	160 ± 10	160 ± 20	160 ± 9	160 ± 9	150 ± 10	170 ± 20	170 ± 10
	CON	230 ± 20	200 ± 20	180 ± 20	160 ± 10	160 ± 10	160 ± 20	160 ± 20	190 ± 40	190 ± 20
**Histidine**	GLU+LEU	68 ± 4	61 ± 2	54 ± 4	57 ± 5	52 ± 4	53 ± 3	49 ± 4	56 ± 3	58 ± 5
	CON	65 ± 5	61 ± 4	62 ± 4	58 ± 3	59 ± 3	60 ± 4	62 ± 5	65 ± 5	70 ± 5
**Glycine**	GLU+LEU	430 ± 40	440 ± 50	380 ± 40	370 ± 40	370 ± 20	380 ± 30	350 ± 30	410 ± 30	400 ± 40
	CON	430 ± 30	430 ± 50	420 ± 50	410 ± 40	400 ± 50	410 ± 50	410 ± 80	440 ± 70	470 ± 70
**Threonine**	GLU+LEU	160 ± 10	120 ± 10	100 ± 10	94 ± 10	88 ± 10	90 ± 7	77 ± 9	88 ± 8	77 ± 8
	CON	140 ± 10	130 ± 10	110 ± 10	91 ± 7	88 ± 7	91 ± 9	100 ± 10	120 ± 20	130 ± 10
**Tyrosine**	GLU+LEU	83 ± 6	70 ± 6	56 ± 7	45 ± 4	45 ± 6	44 ± 5	37 ± 5	42 ± 6	36 ± 4
	CON	84 ± 7	71 ± 7	56 ± 5	51 ± 4	49 ± 4	53 ± 4	47 ± 5	57 ± 4	56 ± 8
**Tryptophan**	GLU+LEU	100 ± 6	110 ± 4	92 ± 7	82 ± 8	81 ± 10	82 ± 8	85 ± 10	85 ± 10	85 ± 10
	CON	110 ± 8	100 ± 10	86 ± 7	76 ± 6	80 ± 7	81 ± 6	93 ± 20	100 ± 10	91 ± 10
**Phenylalanine**	GLU+LEU	85 ± 6	69 ± 5	53 ± 5	50 ± 3	48 ± 5	49 ± 4	46 ± 5	51 ± 5	47 ± 6
	CON	83 ± 5	70 ± 6	61 ± 4	55 ± 2	59 ± 4	59 ± 4	61 ± 7	68 ± 5	68 ± 7
**3-Methyl-histidin**	GLU+LEU	29 ± 2	27 ± 3	26 ± 3	27 ± 2	26 ± 3	26 ± 3	24 ± 3	25 ± 3	25 ± 3
	CON	31 ± 4	27 ± 3	27 ± 2	26 ± 2	25 ± 2	26 ± 2	26 ± 2	28 ± 2	31 ± 2

Mean values ± SEM for plasma amino acid concentrations (μmol/l) before and 60, 120, 150, 180, 210, 240, 270, 360 min after supplementation with leucine and glucose (GLU+CON) or plain water (CON), n = 11. Within a treatment there was no significant differences from resting values. Within a time point, value for GLU+LEU treatment did not differ significantly from the value for the CON treatment.

## Discussion

The results showed that repeated post exercise administration with a mixture of leucine and glucose caused a marked increase in plasma leucine concentration and decreased levels of the other BCAA and methionine. This agrees with the results observed post-exercise in humans after repeated supplementation with glucose together with a mixture of whey, leucine and phenylalanine [17]. A recent study on horses reported a similar effect on the BCAA profile after a single gastric gavage of leucine and glucose in the early recovery period after glycogen depleting treadmill exercise [6]. In the present study, plasma leucine concentrations increased 4-5 fold after supplementation with leucine, which is less than in the study by Urschel *et al. *[6] where plasma leucine concentrations increased 8-10 fold. In that study, the dose was higher (0.3 g/kg BW) and given as a single per oral dose whereas the present study used a lower dose (0.1 g/kg BW) given twice. The administration of leucine at 0 and 4 h post-exercise was chosen since oral administration of leucine stimulates the insulin response for 4 h in horses [21]. Despite this, the decreases in the plasma isoleucine and valine concentrations were in parity to those seen by Urschel *et al. *[6].

Although amino acid concentrations tended to be lower during the post-exercise period in the GLU+LEU treatment, significant changes were only found for the other BCAA and methionine. Decreased concentrations of aromatic amino acids and other free amino acids have been seen in humans after leucine infusion or oral intake of leucine or BCAA at rest [8,10,11] and before exercise [5]. The changes in plasma amino acids seem to follow a certain pattern, with most marked decreases occurring in isoleucine, -55%, methionine -55%, valine -40%, tyrosine -35%, and phenylalanine, -35% [8,10]. In the present study, administration of leucine and glucose was given post exercise and the magnitude of change for isoleucine, valine and methionine were more marked than in humans but similar to the values obtained in horses [6]. The differences in results between studies in humans versus horses could be related to species differences, when the mixture was given (at rest or post exercise), the route of administration, the dosage of leucine as well as the sampling time.

Unlike the previous study in horses [6], the horses in the present study were sampled repeatedly over a period of 360 min post administration, making it possible to study when the changes in amino acid concentrations occurred. The changes in isoleucine occurred earlier (within 60 min after the first leucine supplementation) than the changes in valine and methionine (120 min and 270 min, respectively). This is in agreement with a previous study in humans were methionine levels decreased 45-180 min post ingestion of BCAA [11]. If this is a reflexion in how fast these different amino acids are metabolised in muscle and other tissues (liver, kidney) is not known. Concentrations of plasma free amino acids are often difficult to interpret as they are influenced by many factors such as diet [22], release from tissues (kidney, liver, muscle) as well as red blood cells.

The lack of correlation between the insulin and isoleucine, valine and methionine responses during the recovery period indicates that the changes in these amino acids are not related to the increased insulin response. This is in contrast to results from a study in human athletes were a negative correlation between the insulin response and the concentrations of plasma amino acids was found [17]. In the latter study the athletes were given a mixture of carbohydrates and amino acids (0.1 g/kg/h leucine and 0.1 g/kg/h phenylalanine) after performing glycogen-depleting exercise. The reason for this discrepancy in results between studies could be related to type and dosage of amino acid supplementation and when it was administered, as well as the degree of insulin response. It is not likely that the marked insulin response was too low to influence the plasma amino acid concentration. Studies in horses have also failed to show an increase in glycogen re-synthesis after supplementation with leucine and glucose during the early post-exercise period after glycogen depleting exercise, despite a marked insulin response [7,21]. In addition to this, horses do not show increased insulin sensitivity after glycogen depleting exercise in contrast to humans [23]. Taken together, these findings indicate that horses have a different response to insulin (insulin resistant) compared to humans. In humans an increased glycogen re-synthesis [4,17] is seen after per oral leucine supplementation post exercise as well as an insulin dependent decrease in amino acids [15,17]. Reduced concentrations of isoleucine, valine and methionine in combination with a low insulin response have been reported in humans after intravenous supplementation with leucine during resting conditions [8]. These results suggest that the leucine induced fall in amino acid concentration is independent of insulin, which is supported by the findings from the present study. Although leucine is known to stimulate insulin secretion in humans and horses [5,6], the present study could not find a correlation between the leucine and insulin response. One possible explanation for this could be that there is a dose dependant response in insulin until a certain threshold level is reached, and above this the relationship ceases to exist.

The observed changes in amino acid concentrations are likely related to a direct effect of leucine on protein metabolism as leucine has been shown to stimulate protein synthesis and inhibit protein degradation [19,24,25]. The reduced amino acid concentrations could also be related to an up regulation of the α-ketoacid dehydrogenase complex [26] or an increased uptake of amino acids to muscle or liver. The fact that methionine was decreased could also be related to an increase in glucagon post-exercise as previous studies have shown that glucagon stimulates the uptake of methionine from plasma to the liver [27].

In the present study the horses performed an exercise test in the field in order to deplete muscle glycogen before they were given the two different oral treatments in the recovery period. This exercise test caused alterations in some plasma amino acid concentrations and to a similar degree after both tests. The observed changes in BCAA and aromatic amino acids after exercise and in the recovery period during the CON treatment are in good agreement with a previous study in which horses performed intense standardised exercise on a treadmill to deplete glycogen [28]. Similar increases in the concentrations of leucine and isoleucine, but not in valine, were found in Standardbred trotters after performing intense exercise over a distance of 2000 m [29]. The increase in BCAA and aromatic amino acids after exercise might indicate a certain degree of protein degradation [30]. Amino acid concentrations decreased during the post-exercise period in both treatments but as stated above, the changes were most marked for isoleucine, valine and methionine in the GLU+LEU treatment. This further supports the fact that the decreases seen in these amino acids were related to the treatment and not to an effect of exercise.

## Conclusions

Repeated post-exercise administration of leucine and glucose caused decreased concentrations of isoleucine and valine as well as methionine in a similar pattern as reported in humans. Contrary to man, the changes in these amino acid concentrations did not seem to be related to the insulin response. Further studies are needed to evaluate the mechanism behind the effect of leucine on amino acid metabolism in horses.

## Competing interests

The authors declare that they have no competing interests.

## Authors' contributions

JBR and KNO took equal responsibility for designing the study as well as coordinating the project and apply for funding and drafted the manuscript. The practical experiment was conducted with equal contribution from KNO and JBR with support from BEG. BEG was involved in the sampling of the horses and commented the manuscript. All authors read and approved the final manuscript.
